# Dysfunction of Mesenchymal Stem Cells Isolated from Metabolic Syndrome and Type 2 Diabetic Patients as Result of Oxidative Stress and Autophagy may Limit Their Potential Therapeutic Use

**DOI:** 10.1007/s12015-018-9809-x

**Published:** 2018-04-03

**Authors:** Katarzyna Kornicka, Jenny Houston, Krzysztof Marycz

**Affiliations:** 1 Department of Experimental Biology, The Faculty of Biology and Animal Science, Wroclaw University of Environmental and Life Sciences, Norwida 25, 50-375 Wrocław, Poland; 2 Wroclaw Research Centre EIT+, 54-066 Wroclaw, Poland; 3 PferdePraxis Dr. Med. Vet. Daniel Weiss, Postmatte 14, CH-8807 Freienbach, Switzerland

**Keywords:** Mesenchymal stem cells, Metabolic syndrome, Diabetes, Aging, Senescence, Regenerative medicine, Oxidative stress, Autophagy

## Abstract

Mesenchymal stem cells (MSC) have become a promising tool for therapeutic intervention. Their unique features, including self-renewal, multipotency and immunomodulatory properties draw the worldwide attention of researchers and physicians with respect to their application in disease treatment. However, the environment (so-called niche) from which MSCs are isolated may determine their usefulness. Many studies indicated the involvement of MSCs in ageing and disease. In this review, we have focused on how type 2 diabetes (T2D) and metabolic syndrome (MS) affect MSC properties, and thus limit their therapeutic potential. Herein, we mainly focus on apoptosis, autophagy and mitochondria deterioration processes that indirectly affect MSC fate. Based on the data presented, special attention should be paid when considering autologous MSC therapy in T2D or MS treatments, as their therapeutic potential may be restricted.

## Mesenchymal Stem Cells (MSC)

Progressive obesity, insulin resistance, abnormal cholesterol or triglyceride levels that lead to metabolic syndrome (MS) and finally type 2 diabetes (T2D) are emerging problems in the current endocrinology. As reported by the International Diabetes Federation, 382 million of adults (8.3%) are living with diabetes globally; what is more, in the perspective of next 20 years it is estimated that this number will increase to 592 million [1]. Moreover, type 2 diabetes (T2D) has become the leading cause of death among people under the age of 60. These alarming data are becoming increasingly serious both for medicine and the national health care system. Many strategies have been recently proposed to minimize health-related consequences of metabolic syndrome and diabetes. They involve new drug development, including, e.g., glucagon-like peptide (GLP-1) mimetic, dipeptidyl-peptidase-4 (DPP-4) inhibitors, sodium glucose transporter-2 (SGLT2) inhibitors, but also surgical gastric correction, diet-related therapy, such as calorie restriction and finally mesenchymal stem cells application. Mesenchymal stromal stem cells (MSCs) were first described by Fridenstein and colleges in 1970s [2]. These authors demonstrated that in addition to hematopoietic stem cells (HSCs), also rare, plastic-adherent stromal stem cells resided in the bone marrow that showed the ability to form single colonies, the so-called colony-forming unit fibroblasts (CFU-fs). Fridenstein and colleagues observed that this cell population expanded in culture, but more importantly, they demonstrated that MSCs had the ability to differentiate into mesoderm-derived tissue and played an important role in controlling the hematopoietic niche [3]. Thirty-two years later, Zuk and co-workers described for the first time cell population in adipose tissue termed processed lipoaspirate (PLA) cells, which were isolated from human lipoaspirates and, like MSCs, differentiated towards osteogenic, adipogenic, myogenic, and chondrogenic lineages [4]. These physiological features of MSCs are consistent with the general knowledge regarding the regulation of tissue homeostasis. Since Friedenstein, the knowledge about MSC physiological nature has been extensively investigated in the fields of human and veterinary medicine as well as biology [5–14]. Unique cytophysiological properties of this stem cell population have led to developing a concept, in which their clinical application is consequently implemented. Both MSCs of bone marrow (BMSCs) and adipose tissue (ASCs) have become the most frequently used sources of progenitor cells in the field of tissue engineering and/or regenerative medicine. This population of stem cells, because of their multipotent character, anabolic activity and immunomodulatory effect as well as the ability to differentiate into insulin-producing cells, is a promising candidate in the field of endocrine disorders, including type 2 diabetes and metabolic syndrome [15–17]. Here, we analyze physiological characteristics and deterioration of MSCs in metabolic syndrome and diabetes in the context of their potential clinical application.

## MSC and Membrane-Derived Vesicles (MVs)

The pro-regenerative nature of MSCs was excellently explained and described for the first time by Ratajczak and colleagues, who proposed membrane-derived vesicles (MVs) as a carrier for a wide range of growth factors secreted into the intercellular space, thereby enhancing regenerative processes [18–20]. Moreover, it has been proposed that MVs could be internalized by the donor cell wall or taken up by neighboring cells and ultimately improve intercellular signaling. Therefore, the improvement of intercellular communication is believed to be the initiator of the regenerative process of damaged cells and tissues [21, 22]. For this reason, MVs in addition to MSCs are strongly considered as the candidate to initiate the regenerative process. What is more, MVs have been detected in the circulation and in organs during various diseases, including diabetes and metabolic syndrome [23]. Patients suffering from those disorders, have different cellular MV patterns and those MVs contribute to the development of diabetic macrovascular and microvascular complications. Changes in MV number and composition may serve as potential biomarkers for diagnostic including diabetes and MS.

Bearing in mind the fact that both MS and T2D are characterized by chronic inflammation, searching for systemic, anti-inflammatory agents seems to be fully reasonable. It is very possible that inflammation is the most important factor leading to β-cell dysfunction and eventually death. Hyperglycemia, in combination with altered lipid metabolism (so-called “glucolipotoxicity”), induces local cytokine production and affect the accumulation of oxidative stress factors. Considering the above fact, MSCs and MVs are promising candidates for an effective delivery of anti-inflammatory agents. It was reported that MSCs exerted an immunomodulatory effect through abundant synthesis and secretion of anti-inflammatory cytokines, such as IL-1Ra, IL-10 or transforming growth factor beta (TGF-β) [15]. However, it is not clear how anti-inflammatory cytokines could be transferred to donor β-cells or whole islets. It seems that this direction is fully rational and may bring future advances in MS and T2D treatment. However, it still needs to be explained whether MSCs or MSC-derived MVs obtained from MS or T2D patient will require pharmacological treatment before clinical application. It was shown that MSCs of obese, MS, and T2D patients suffered from increased apoptosis and limited multipotency [16, 24]. These facts should be considered before both MSCs and MVs will find their application in endocrinology practice.

## Aging and Senescence of MSC from MS and Diabetic Individuals

Although MSCs can be found in multiple organs and tissues, e.g., bone marrow, adipose tissue, dental pulp and Wharton’s jelly, the final cell yield after isolation is rather low. This creates a demand for prolonged *in vitro* culture expansion to millions of cells required for therapy. Time of expansion strongly depends and correlates with donors’ age, genetic makeup and clinical history [25–27]. It was shown that aged MSCs suffered from multiple deteriorations that strongly diminished their therapeutic utility [26].

Senescence and aging of MSCs has been well known and described in a number of studies that used MSCs from multiple sources, including dental pulp [28–30], adipose tissue [8, 31], bone marrow [26, 32], cord blood [33] and endometrium [34]. Senescence activation pathway was correlated with different stress stimuli, such as oxidative stress [35], heat shock or chemotherapeutic agents [36, 37]. It has been demonstrated that aged MSCs accumulate excessive amount of oxidative stress factors and simultaneously suffer from decreased antioxidative defense. Moreover, they exhibit decreased proliferation rate, higher susceptibility to apoptosis and decreased multilineage differentiation potential, which strongly limits their therapeutic value [8, 11, 31, 38].

It was shown, that MS may affect stem cell pool, as the decreased level of CD34 and endothelial progenitor stem cells was associated with MS progression. In normal conditions, MSCs usually remain in a quiescent and undifferentiated state in their niches (pools). Although “alarms” like proinflammatory cytokines, such as INF-α or IL-6, and growth factors like GM-CSF are able to mobilize MSCs and induce their proliferation and homing [39, 40]. Considering the increased caloric uptake in T2D and MS patients, higher secretion of proinflammatory factors may contribute to constant emission of “alarms” and MSC mobilization [41, 42]. Depletion of MSC pool contributes in turn to irreversible impairment of tissue regeneration.

The proinflammatory environment of adipose tissue negatively affects MSC stemness. It was found that MSCs isolated from EMS (equine metabolic syndrome) individuals suffered from many deteriorations, including decreased proliferation rate, clonogenic potential and increased population doubling time [24]. Moreover, MS affected the expression of MSC surface antigens, as substantial reduction of CD90, CD105 and CD73 levels was observed. Decreased expression of CD90 was also observed by Koci et al. [43], although in patients suffering from T2D. Moreover, a study on the diet-induced obese mice showed decreased CD105 expression, which altered the properties of adult stem cells [44]. Interestingly, MS cells were characterized by increased expression of CD44, a cell surface glycoprotein working as an immune cell receptor involved in inflammatory cell activation. Research conducted by Kodama and colleagues [45–47] revealed that CD44 was upregulated in white adipose tissue of obese, diabetic mice and humans. Interestingly, CD44 knockout mice fed a high fat diet, did not develop obesity or T2D. The upregulation of CD44 led to migration and infiltration of activated immune cells, increasing the inflammation in adipose tissue; in addition, it was also confirmed that MS adipose tissue was enriched in macrophages secreting IL-1, IL-6 and TNF-alpha. Moreover, reduced expression of MVs was observed in those cells, which could have contributed to their decreased therapeutic potential and utility. Both MS and T2D strongly affect MSC morphology, including actin cytoskeleton organization. MS cells display typical senescence features, including enlarged nucleus and widely spread cellular body, the so-called “fired egg shape” [24]. The increase in the cell population doubling time and changes in their morphology (larger cell size and structural complexity) are also associated with increased apoptosis of MSCs from obese individuals [48].

Susceptibility to apoptosis and increased senescence are another features of MS and T2D stem cells. The upregulation of p53, p21 and BAX was observed in those cells. Moreover, higher levels of anti-apoptotic protein Bcl-2, which prevents activation of caspase cascade and inhibits the release of cytochrome c from mitochondria were also noted. Accordingly, excessive accumulation of senescence-associated β-galactosidase, increased number of dead cells and decreased Ki67 expression may further contribute to the reduction of MSC stemness. Although in the study conducted by Nawrocka et al. [49], these pathological changes caused by T2D appeared to be abolished by the supplementation with basic fibroblast growth factor (bFGF). The addition of bFGF to culture media significantly improved MSC proliferation and decreased apoptosis rate.

Although stem cells retain their self-renewal abilities both in vivo and in vitro, oxidative, inflammatory environment of adipose tissue may contribute to their lower stemness status. Differentiation capacity of MSCs seems to vary depending on cell source, age and patient health status. Most attention has been paid to osteo- and chondrogenic properties of MSCs, because of their potential use in tissue engineering where scaffolds combined with stem cells are applied in damaged organ regeneration. Although MS and T2D contributes to decreased multipotency of MSCs by generating advanced glycation products (AGEs), oxidative stress and inflammation, which can suppress proliferation, induce apoptosis and increase the production of intracellular reactive oxygen species (ROS). Increased apoptosis and ROS accumulation may be partially responsible for the reduced differentiation potential observed in MS cells. It was found during the osteogenic differentiation process that the expression of bone BMP-2 as well as Coll-1 at the mRNA level was significantly downregulated in MS, whereas the expression of RUNX2, OCN and ALP was comparable with the control group [50]. Similar results were obtained during chondrogenic differentiation, as MS cells presented decreased expression of vimentin, decorin and Sox9 [51]. The study conducted by Wu et al. [44] showed that MSCs from obese donors exhibited increased adipogenic and osteogenic differentiation, but decreased chondrogenic capacity. Similarly, MSCs isolated from diabetic patients showed reduced osteogenic differentiation and chemokine CXCL12 downregulation [43].

Metabolic syndrome strongly affects self-renewal and differentiation capacities of MSCs. Thus, their application in clinical practice requires further research and analysis in the context of their safety. The innovative approach is to “rejuvenate” these cells before their clinical use with different chemicals.

## Oxidative Stress, Autophagy and Mitophagy in MSCs

Living cells are continuously exposed to the harmful effect of exogenous or endogenous reactive oxygen species (ROS). These highly reactive molecules, radicals and non-radicals, have the ability to capture electrons from molecules they come in contact with, including proteins and nucleic acids, leading in consequence to cell damage. Moreover, oxidative stress may cause non-specific, post-translational protein modifications, leading to aggregate formation. It is commonly known, that the mitochondrial respiratory chain is the main source of ROS in cells. Complexes I and III are most susceptible to electron leakage, resulting in H_2_O_2_ formation. Moreover, in certain circumstances, the electron flux is intensified, as in increased energetic demand during endurance exercises. On the other hand, mitochondrial function may decrease during aging or degenerative and metabolic diseases. Hence it is crucial for cells to have a highly effective oxidative defense. Superoxide dismutase (SOD) and glutathione peroxidases are the most important enzymes involved in free radical scavenging. An efficient antioxidant system is also necessary to cope with reactive nitric species (RNS) generated by the reaction between O_2_ and nitric oxide (NO) [52]. Similarly to ROS, excessive accumulation of RNS leads to irreversible damage to biomolecules [53].

Mitochondrial ROS production and oxidation of mitochondrial lipids have been shown to play a role in inducing autophagy. Autophagy (or self-eating) was first described by Christian de Duve in 1963 as a lysosome-mediated degradation process for non-essential or damaged cellular constituents [54, 55]. It is an evolutionarily conserved process, in which cells engulf a portion of the cytoplasm and organelles into double-membraned vesicles called autophagosomes, which later fuse with lysosomes for the degradation of enclosed materials [56]. Generation of the pre-autophagosomal structure requires the beclin-1–class III PI3K (phosphoinositide 3-kinase) complex as well as generation and insertion of LC3 (light chain 3)-II into the autophagosomal membrane [57]. Fusion of autophagosomes to lysosomes may be mediated by Rab7 and a lysosomal transmembrane protein, LAMP-2 [58]. Upon autophagosome formation and fusion of its outer membrane with the lysosome membrane, the contents as well as the inner membrane of autophagosomes are degraded to generate amino acids and other cellular building blocks for recycling by the cell. In addition to its clearing function in response to energy or nutrient deficiency, autophagy is also a quality control mechanism for protein and organelles, including mitochondria [59]. Activation of autophagy subsequently leads to damaged proteins and impaired organelle clearance, allowing to maintain cellular homeostasis and remodeling during development. However, the function of autophagy in MSCs is still poorly understood. Available data indicate the importance of autophagy in self-renewal, differentiation and pluripotency of stem cells [60]. Elimination of damaged, dysfunctional proteins and organelles seems to be essential for maintaining pluripotency of adult stem cells during the quiescent state [61].

Besides the main function of energy production, mitochondria are also able to turn on and tune autophagy. Excessive accumulation of ROS leads to impairment of mitochondria structure and function, which in turn triggers a selective process of mitochondria self-removal called mitophagy. It has been proposed that upon nutrient deprivation, mitochondria protect themselves from degradation by promoting fusion and inhibiting fission events. It is only after long-term starvation that mitochondria undergo fragmentation and are eventually removed by mitophagy [62].

The importance of autophagy in MSC physiology has been established in several studies. Research conducted by Sanchez et al. [63] revealed that stromal cells utilized autophagy for survival and secreted anti-apoptotic factors under nutrient-deprived conditions that can occur in solid tumors. It was also demonstrated that the apoptosis of bone marrow MSCs under hypoxia was regulated by autophagy via the AMPK/mTOR pathway [64]. Moreover, Gao and colleagues [65] reported that the level of autophagy regulated CD4+ T cell immunosuppression through MSCs by affecting TGF-β1 secretion, thereby providing a novel method for improving the therapeutic efficacy of MSCs by autophagy activation.

Emerging body of evidence indicates the role of autophagy in the pathogenesis of MS and T2D [66]. High concentration of glucose and reduced insulin sensitivity lead to imbalances in oxidative defense within the cell, resulting in ROS-mediated damage in both disorders. Thus, autophagy disorders cause the accumulation of deteriorated proteins and organelles. It appears that the accumulation of defective mitochondria contributes to the reduced insulin secretion by β-cells [67]. Moreover, autophagy may play a role in maintaining intracellular insulin content by accelerating insulin degradation rate in β-cells [68]. The occurrence of autophagy in diabetic tissues positively correlates with excessive accumulation of ROS, as these two processes are tightly correlated. It has been shown that ROS are produced in various tissues under diabetic conditions [69]. In the diabetic state, hyperglycemia and subsequent ROS production decrease insulin expression and secretion and cause apoptosis [70–72]. In addition, the cells are sensitive to ROS due to the relatively low expression of antioxidant enzymes, such as SOD and glutathione peroxidase [73]. Therefore, it is likely that antioxidant supplementation can protect β-cells against glucose toxicity; this notion was supported by the study of Kaneto et al., who demonstrated that antioxidant treatment retained glucose-stimulated insulin secretion and ameliorated glucose tolerance in obese diabetic C57BL/KsJ-db/db mice [74].

**Fig. 1:**
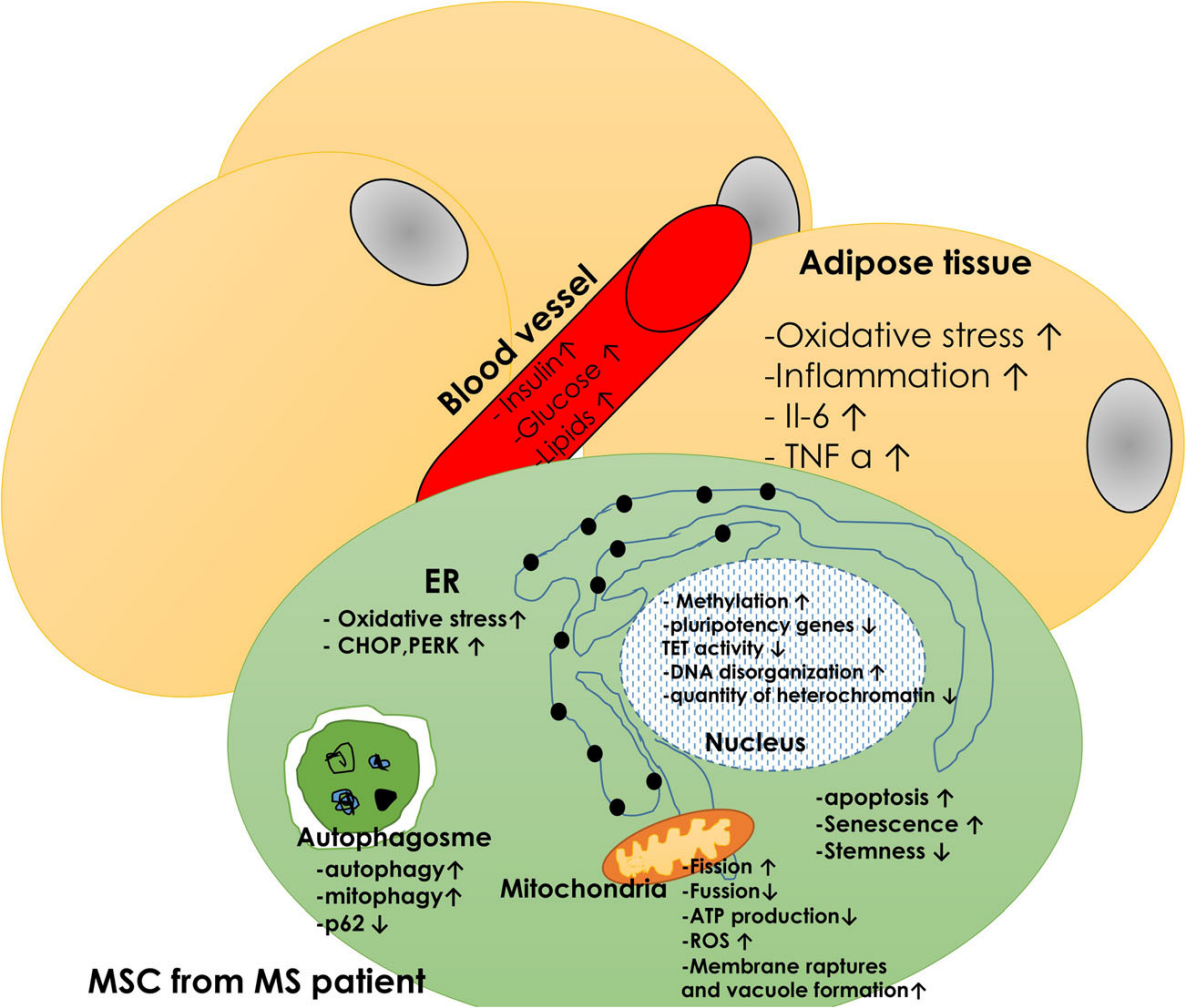
Mesenchymal stem cell dysfunction in metabolic syndrome

Although ROS and autophagy are being investigated in T2D and MS in various tissues, these processes in diabetic MSCs are still not well explained. It is well known that ROS accumulate with age and their excessive levels contribute to decreased therapeutic potential of aged MSCs [8, 26, 32]. Excessive amounts of ROS in MSCs can impair self-renewal, proliferation and differentiation potential [75, 76]. Thus, it is tempting to speculate that autophagy in MSCs may help to overcome harmful effects of ROS; especially that MS and T2D stem cells are characterized not only by ROS accumulation and decreased SOD activity, but also by mitochondria deterioration [24, 49–51]. High frequency of mitochondrial morphological abnormalities, including disarrayed formation of cristae and vacuoles were observed in MSCs isolated from MS individuals. Moreover, PGCα and Parkin expression was significantly decreased in those cells. This fact may contribute to the deterioration of MS_MSC_ osteogenic differentiation, as it has been shown that the transition towards a more oxidative phenotype and increased mitochondrial mass is required to initiate this process [77]. Furthermore, it was reported that excessive ROS inhibited osteogenesis during chondrogenesis and adipogenesis [78]. Hence the hypothesis that ROS and oxidative stress must decrease in order to allow for osteogenic differentiation to proceed seems reasonable. It was shown that MS_MSC_ exhibited decreased osteo- and chondrogenic potential with simultaneous increased rate of mito- and autophagy. The autophagic flux in these cells was probably activated by excessive amount of ROS and damaged mitochondria, which led to nutrient and ATP deprivation. The increased expression of autophagy-related genes, including Beclin-1, LC3 and LAMP2 was observed in MS_MSC_ under controled, chondrogenic and osteogenic conditions. Moreover, p62 concentration was decreased, suggesting that increased autophagy might be a cytoprotective mechanism used by these cells to survive in the inflammatory microenvironment of adipose tissue and to differentiate *in vitro*. The impaired remodeling of mitochondrial network caused by decreased biogenesis and mitophagy results in the accumulation of damaged mitochondria in MS_MSC_, which in turn triggers autophagic turnover to generate ATP necessary for effective differentiation [50]. MSC deterioration in metabolic syndrome, including ROS, mitochondria damage and epigenetic alternations is visualized in Fig. 1.

Similar results were obtained during chondrogenic differentiation of MS_MSC_, as reduced number of mitochondria with a parallel higher expression of lysosomal-associated membrane protein 2 (LAMP2) were observed. Those cells presented slightly impaired chondrogenic differentiation potential, but high auto- and mitophagy allowed to maintain multipotency capacity. Beclin1 and LC3 were downregulated in the native, nonchondrogenic culture, which supports the thesis that autophagy may be a fundamental mechanism that allows MSCs to preserve their multipotency. Moreover, autophagy may be caused by the increased stress of endoplasmic reticulum characteristic of MS_MSC_ [51]_._

The past decade has witnessed a significant interest in stem cells and autophagy [79, 80]. However, research in these areas is in its infancy. Only few studies have investigated how metabolic syndrome or diabetes affect cytophysiological characteristics of mesenchymal stem cells, such as mitochondria metabolism, autophagy and its role in the differentiation. The potential use of autophagy modulation in optimizing MSC differentiation may improve therapeutic potential of these cells. However, this phenomenon requires further investigation and analysis in the context of MSC safety.

## Rejuvenation of Deteriorated cells *In Vitro*

Searching for effective strategy able to restore stemness and reverse aged phenotype of MSC is crucial while considering their clinical application. Multiple chemicals have been tested *in vitro* in order to improve viability and differentiation potential of impaired MSC. One of the most effective substance in attenuation of stem cell senescence is resveratrol (RES). It was shown to decrease oxidative stress, reduce apoptosis, enhance proliferation and paracrine activity of MSC [12, 81]. It was also proved, that sirtuin 1 overexpression delays senescence in MSCs that have undergone prolonged culturing and sustain their adipogenic and osteogenic potential [82]. Furthermore, targeting mTOR pathway with rapamycin reversed the senescent phenotype and improved immunoregulation of MSCs [83]. Recent data indicated that algae extract may be beneficial in reversing ROS induced damage and mitochondrial impairment of MSC derived from metabolic syndrome individuals [75]. Authors, have demonstrated that *Spirulina platensis* extract enhanced viability, suppressed senescence, decreased ROS levels and improved mitochondrial membrane potential of those cells. Furthermore, effectiveness of *Spirulina platensis* was confirmed *in vivo*. Algae supplementation decreased weight and improved insulin sensitivity in metabolic syndrome animals. To summarize, application of substances able to reduce oxidative stress in MSCs may in consequence increase the possibility of therapeutic application of this cells.

## Concluding Remarks

The age and health of patients strongly affects the status of MSCs. The cells isolated from T2D or MS patients are characterized by increased apoptosis, autophagy, ROS accumulation and mitochondria deterioration. The autophagic flux observed in these cells may be a protective mechanism that provides energy and building blocks to restore cellular homeostasis and control oxidative damage. However, to date, only a few studies have shown the role of autophagy in mesenchymal stem cells in metabolic disorders. Based on presented data, therapeutic application of MSC isolated from MS or T2D patients may be limited due to their dysfunctionality.
